# Chromatographic Analysis and Anti-Oxidative Property of Naoxinqing Tablet, a Proprietary Preparation of *Diospyros Kaki* Leaves

**DOI:** 10.3390/molecules24061101

**Published:** 2019-03-20

**Authors:** Magdy Kazzem, Yu-Ting Sun, Mitchell Low, Sai Wang Seto, Dennis Chang, Samiuela Lee, Harsha Suresh, Cheang S. Khoo, Alan Bensoussan, Hosen Kiat

**Affiliations:** 1 NICM Health Research Institute, Western Sydney University, Penrith, NSW 2751, Australia; M.Kazzem@westernsydney.edu.au (M.K.); yu-ting.sun@westernsydney.edu.au (Y.-T.S.); D.chang@westernsydney.edu.au (D.C.); 17271790@student.westernsydney.edu.au (H.S.); A.Bensoussan@westernsydney.edu.au (A.B.); 2 National Measurement Institute, North Ryde, NSW 2113, Australia; sam.lee@measurement.gov.au; 3 School of Medicine, Western Sydney University, Penrith, NSW 2571, Australia; hosen.kiat@chi.org.au; 4 Wentworth Institute, Surry Hills, NSW 2010, Australia; khoo2031@gmail.com; 5 Faculty of Medicine, University of New South Wales, Kensington, NSW 2052, Australia; 6 Faculty of Medicine and Health Sciences, Macquarie University, Macquarie Park, NSW 2113 Australia

**Keywords:** Naoxinqing, UPLC MS/MC analysis, anti-oxidative, endothelial cells, reactive oxygen species

## Abstract

The Naoxinqing (NXQ) tablet is a standardised proprietary herbal product containing an extract of persimmon leaves (*Diospyros kaki*) for the management of cardio- and cerebrovascular diseases. Although previous reports suggested that the efficacy of NXQ is at least partly mediated by its anti-oxidative property, the anti-oxidative effect of the major components of NXQ has not been studied systematically. For quality control purposes, only analytical methods limited to 3 marker analytes have been reported, the extent to which the other components affect efficacy has not been explored. In this study, we developed an ultra-performance liquid chromatography-tandem mass spectrometry (UPLC MS/MS) method for the identification of seven analytes (kaempferol-3-*O*-glucoside (astragalin), quercetin-3-*O*-galactoside (hypericin), quercetin-3-*O*-glucoside (isoquercitin), kaempferol, 3,4-dihydroxybenzoic acid (protocatechuic acid), and furan-2-carboxylic acid (pyromucic acid) and quercetin) in the NXQ. This is the first method reported and validated for the quantification of the seven major secondary metabolites in NXQ. The results for the quantified analytes were then compared in 15 different batches of NXQ. The variation observed in the seven components highlights the need to quantify key bioactive components to ensure product consistency. Radical scavenging activity and abundance was used to rank the analytes. The anti-oxidative effects of NXQ were examined using cultured human vascular endothelial cells (EA.hy926). Corrected 2,2-di(4-tert-octylphenyl)-1-picrylhydrazyl (DPPH) activity results revealed that quercetin and kaempferol have the strongest anti-oxidant capacity in the extract. Both quercetin and kaempferol significantly inhibited the hydrogen peroxide (H_2_O_2_)-induced EA.hy926 cell injury and intracellular reactive oxygen species (ROS) generation. In conclusion, we established and validated an UPLC-MS/MC method for the analysis of major bioactive components in the NXQ and demonstrated that its anti-oxidative property may play a critical role in cerebrovascular protection.

## 1. Introduction

Strokes are one of the major causes of death and adult disability worldwide [1]. The underlying pathophysiology of a stroke is highly complicated, consisting of impairments of multiple signaling pathways, and numerous pathological processes such as acidosis, glutamate excitotoxicity, calcium overload, cerebral inflammation and reactive oxygen species (ROS) generation [2]. The current therapeutic approach for stroke treatment focuses on neuroprotection, thrombolysis and surgical clot removal [3]. Despite promising results from the studies of numerous interventions in animal models of stroke, translation of these findings to the bedside has been disappointing [4]. So far, recombinant tissue plasminogen activator (tPA) is the only approved therapy available for ischemic stroke [5]. However, tPA has a very narrow therapeutic window, which can only be given up to 6 hours after the onset of a stroke and, therefore, only reaches less than 10% of stroke patients [6].

Chinese herbal medicine (CHM) has a long history of clinical use for stroke prevention, treatment and rehabilitation [7]. Data from numerous clinical studies have demonstrated the potential benefits of Chinese herbs with a high level of flavonoids and sulphur compounds in a stroke [8,9]. Recent cerebral ischemia clinical and large population-based epidemiological studies have demonstrated potential benefits of CHMs for an ischemic stroke [8,10].

The Naoxinqing (NXQ) tablet is a patented CHM preparation containing a standardised aqueous extract of *Diospyros kaki L.f.* (Ebenaceae) (Shi Ye in Chinese) leaves. In China, NXQ has been clinically used for many years in the treatment of arthrosclerosis-related diseases especially for coronary heart disease and stroke [11,12,13,14,15]. Early evidence exists to demonstrate potential clinical benefits of NXQ in stroke patients with cerebral atherosclerosis, transitory ischemia syndrome (TIS), cerebral thrombosis sequela, apoplexy sequel and cerebral embolism, with few side effects [16]. However, these claims have not been thoroughly validated in large-scale double blind clinical trials. Additionally, the mechanisms of action underlying the clinical effects of NXQ is largely unknown although a few studies report multiple pharmacological effects of NXQ including neuroprotection, blood pressure lowering, thrombosis inhibition and microbial inhibition [11,12,13]. In a recent study, several undescribed triterpene saponins isolated from *Diospyros kaki* leaves have been shown to inhibit hydrogen peroxide-induced damage in human dopaminergic neuroblastoma cells [17], suggesting its neuroprotective property. In a mouse model of Alzheimer’s disease, flavonoids extracted from the leaves of Diospyros kaki reversed memory impairment and rescued synapse loss *via* modulation of RhoA-GPTase activity [18]. Furthermore, Bei et al. (2004) demonstrated that NXQ reduced oxidative stress in NG108-15 cells by improving redox imbalance and inhibition of apoptosis, highlighting its anti-oxidative property [11].

A number of phytochemical studies have shown that triterpenoids and phenolic compounds are present in the leaves of *Diospyros kaki* [14,19,20]. Given the significant antioxidant property of NXQ for stroke prevention, treatment and recovery, the bioactive antioxidants in NXQ will be of particular relevance for quality assurance (QA) and efficacy. For example, Hossain et al. (2017) showed that bioactive compounds and their antioxidant properties were greatly affected by pre-treatment and extraction conditions, such as harvesting season, drying temperature and extraction time [21]. The bioactive flavonoids including quercetin and kaempferol have been suggested to be the main therapeutic constituents of NXQ [13] even though other bioactive components may also play a role [22] with 41 compounds identified in the extract [23]. Quercetin and kaempferol are closely related, with quercetin possessing one more hydroxyl group than kaempferol. Both are flavonoids, a chemical class known for their radical scavenging and inhibition of reactive oxygen species (ROS) activity in the body, thereby alleviating oxidative damage in neural cells [13].

Although quercetin and kaempferol may be the major actives, this has not been systematically tested and the contribution of the other 39 reported polyphenols [23] has not been explored. There is a lack of validated analytical methods to quantify the other major component, with only up to 3 analytes being reported [24]. In this study we established an ultra-performance liquid chromatography-tandem mass spectrometry (UPLC-MS) method for quantitation of several major analytes including kaempferol-3-*O*-glucoside (astragalin), quercetin-3-*O*-galactoside (hypericin), quercetin-3-*O*-glucoside (isoquercitin), kaempferol, 3,4-dihydroxybenzoic acid (protocatechuic acid), furan-2-carboxylic acid (pyromucic acid) and quercetin in NXQ. Furthermore, the concentrations of the analytes were tested in 15 batches of NXQ. The free radical scavenging property of the antioxidant components of the NXQ were assessed and ranked by 2,2-di(4-tert-octylphenyl)-1-picrylhydrazyl (DPPH) and 2,2′-azino-bis(3-ethylbenzthiazoline-6-sulphonic acid (ABTS) assays. Finally, the anti-oxidative property of the top two ranked free radical scavengers was evaluated using cultured human endothelial cell line, EA.hy926.

## 2. Materials and Methods

### 2.1. Materials

Kaempferol-3-*O*-glucoside, quercetin-3-*O*-galactoside, quercetin-3-*O*-glucosideand kaempferol were obtained from Zhongxin Pharmaceuticals (Tianjing, China). Furan-2-carboxylic acid and quercetin were obtained from Sigma-Aldrich (Castle Hill, New South Wales, Australia) and 3,4-dihydroxybenzoic acid was obtained from Merck (Frenchs Forest, New South Wales, Australia).

Liquid chromatography (LC)-grade methanol (MeOH) was supplied by Mallinckrodt Chemicals Ltd. (Derbyshire, United Kingdom). N_2_ gas (high purity (HP) grade) and Ar gas (ultrahigh purity (UHP) grade) were supplied by BOC gases (Sydney, New South Wales, Australia). Ethanol and formic acid (AR grade) were from Merck (Frenchs Forest, New South Wales, Australia). 

The NXQ tablets were provided by Hutchison Whampoa Guangzhou Baiyunshan Chinese Medicine Co., Ltd. (Guangzhou, China). Analytical method development and validation was performed on NXQ pills from Batch A-2. The developed method was then used to analyse 15 batches of NXQ pills.

Hydrogen peroxide (H_2_O_2_), dimethyl sulfoxide (DMSO) and trypan blue were purchased from Sigma-Aldrich (St. Louis, MO, USA). Dulbecco’s Modified Eagle’s Medium Ham’s F-12 (DMEM/F12) (1:1 Mix) with l-glutamine was purchased from Lonza (Morristown, NJ, USA). Foetal bovine serum (FBS) and penicillin and streptomycin (PS) were purchased from Gibco Life Technologies (USA). 3-(4,5-di-methylthiazol-2-yl) 2,5-diphenyltetrazolium bromide (MTT) was purchased from Astral Scientific (Taren Point, New South Wales, Australia). The cellular ROS/Superoxide detection assay kit was purchased from Abcam (Cambridge, UK). 

### 2.2. Preparation of Stock Solutions and Working Standards

A mixed stock standard solution containing kaempferol-3-*O*-glucoside (0.53 µg mL^−1^), quercetin-3-*O*-galactoside (0.17 µg mL^−1^), quercetin-3-*O*-glucoside (0.41 µg mL^−1^), kaempferol (0.39 µg mL^−1^), 3,4-dihydroxybenzoic acid (0.12 µg mL^−1^), furan-2-carboxylic acid (0.27 µg mL^−1^), and quercetin (0.49 µg mL^−1^) was prepared in LC grade MeOH. 

On the day of the analysis, working standards were prepared by diluting the stock with MeOH to give 10, 25, 50, 75 and 100% of the stock concentration. Curve fitting was obtained using the least-squares method.

### 2.3. Extraction of the Naoxinqing (NXQ)

Approximately 10 mg of the ground and sieved sample was accurately weighed into a pre-weighed aluminium weigh boat using an analytical micro balance. Approximately 7 mL of LC-grade MeOH extraction solvent was added and the flask capped and sonicated for 30 min. The solution was allowed to cool to room temperate before making up to volume (10 mL) with MeOH. The sample was mixed by inversion several times and filtered through a 0.20 µm PVDF filter into a 2 mL LC vial using a 5 mL syringe for LC analysis.

### 2.4. Ultra Performance Liquid Chromatography-Electrospray Ionization-Tandem Mass Spectrometry (UPLC-ESI-MS/MS) Instrumentation

A Waters Inc. (Milford, MA, USA) Acquity UPLC with a Xevo TQ MS tandem MS detector with electrospray ionization (ESI) was used for the analysis. Data processing was performed using the Waters (Milford, MA, USA) MassLynx V4.1 SCN 714 software. The analytical column was a Waters (Milford, MA, USA) ACQUITY UPLC BEH reversed phase C18 column (2.1 × 50 mm, 1.7 µm), with a Waters (Milford, CO, USA) reversed-phase C18 guard column.

### 2.5. UPLC-ESI-MS/MS Conditions

The flow rate was set at 0.30 mL min^−1^, injection volume 1 µL and column temperature 22 °C. The gradient mobile phase consisted of aqueous formic acid (0.1% *v*/*v*) (A): MeOH (B). The initial solvent composition was 70% (A): 30% (B), changing to 45% (B) over 15 min and then 100% (B) over 2 min and maintained at this composition for 3 min before equilibrating at the initial composition for 5 min before the next injection.

The individual standards (50 µg mL^−1^) in MeOH were infused directly into the ESI interface to optimise ionisation and ion transmission conditions. The N_2_ nebulisation gas temperature was 300 °C and the cone voltages were set accordingly for each analyte as shown in Table 1. The polarity (+) or (−) of the ESI mode was determined at the same time. Direct infusion also determined the optimal MS/MS conditions by selecting the appropriate precursor ion (*m*/*z*) for each analyte and the collision energy of the Ar gas required to induce fragmentation to produce at least two product ions. 

### 2.6. Method Validation

To determine analyte recoveries a mixed spiking solution containing the same ratio of concentrations of the analytes as in the sample was prepared. The spiking solution contained kaempferol-3-*O*-glucoside (5.3 µg mL^−1^), quercetin-3-*O*-galactoside (1.7 µg mL^−1^), quercetin-3-*O*-glucoside (4.1 µg mL^−1^), kaempferol (3.9 µg mL^−1^), 3,4-dihydroxybenzoic acid (1.2 µg mL^−1^), furan-2-carboxylic acid (2.7 µg mL^−1^), and quercetin (4.9 µg mL^−1^). This solution was used for the 50, 100 and 200% analyte spike levels where 50, 100 and 200 µL of the spiking solution was dispensed into the pre-weighed sample (~10 mg) and the solvent was allowed to evaporate by standing for ≥3 h before extraction and analysis of the extract. The recovery for each spike level was determined with 7 replicates as well as the unspiked sample to give a total of 28 samples. 

The limit of detection (LOD) and the limit of quantification (LOQ) of the method were determined from the standard deviation (SD) of replicate analyses (*n* = 7). All the experiments and analyses were performed by a single operator using same equipment to avoid any interlaboratory variations.

### 2.7. 2,2-Di(4-Tert-Octylphenyl)-1-Picrylhydrazyl (DPPH)Scavenging Activity

The DPPH radical scavenging capacity was based on the method reported by Zhang et al. [25]. The reference standards were prepared to 1000 µM and diluted (1:2) to 7.8 µM, with all dilutions being assayed in triplicate. The NXQ was prepared at 5 mg mL^−1^ and diluted (1:2) to 0.08 mg mL^−1^ with all dilutions being assayed in triplicate. All reagents were prepared in 80% aqueous methanol, 150 μL of the DPPH reagent (62.5 μM) was added into each microtiterplate well and then 50 μL of either working, sample or blank to make a total volume of 200 μL. To correct for sample absorbance (i.e., absorbance not due to DPPH), sample blanks were prepared by adding 150 μL of 80% aqueous methanol to the well and 50 μL of sample. The plate was vortexed at 700 rpm for 30 min in the dark prior to measuring absorbance at 515 nm. The sample antioxidant DPPH scavenging capacity is reported as the EC50 (µM).

### 2.8. 2,2′-Azino-Bis(3-Ethylbenzthiazoline-6-Sulphonic Acid(ABTS)Scavenging Activity

The ABTS scavenging assay was based on the method reported by Cai et al. [16]. The ABTS radical cation was generated by reacting 7 mM ABTS and 2.45 mM potassium persulphate after incubating at room temperature in dark for 16 h. The ABTS solution was then diluted with phosphate buffered saline (PBS) to an absorbance of 0.700 ± 0.050 at 734 nm. The reference standards were prepared to 1000 µM and diluted (1:2) to 7.8 µM, with all dilutions being assayed in triplicate. The NXQ was prepared at 5 mgmL^−1^ and diluted (1:2) to 0.08 mgmL^−1^ with all dilutions being assayed in triplicate. 10 µL of the samples (or blank) were mixed with 190 µL of ABTS solution. To correct for sample absorbance (i.e., absorbance not due to ABTS), sample blanks were prepared by adding 190 μL of PBS to the well and 10 μL of sample. The plate was vortexed at 700 rpm for 5 min in the dark prior to measuring absorbance at 734 nm. The sample antioxidant ABTS scavenging capacity is reported as the EC50 (µM).

### 2.9. EA.hy 926 Cell Culture

The permanent human endothelial cell line EA.hy926 was originally derived from a human umbilical vein obtained from ATCC (USA). In this study, cells were grown in DMEM/F12 (1:1 mix) supplemented with 10% foetal bovine serum (FBS), 1% l-glutamine and 1% penicillin-streptomycin in a humidified atmosphere of 5% CO_2_ at 37°C. During cell culture, the medium was changed every 3 days until the cells reached 80–90% confluence. To assess the effects of NXQ on EA.hy926 cells, the cells were treated with increasing concentrations of quercetin (0.1–500 µg mL^−1^) or kaempferol (0.1–500 µg mL^−1^) for 2 h followed by H_2_O_2_ (0.5 mM) or a vehicle for 24 h unless stated otherwise. 

### 2.10. Measurement of Intracellular Reactive Oxygen Species (ROS) Level

Intracellular ROS level was evaluated using the cellular ROS/superoxide detection assay kit (Abcam, UK) as previously reported [26]. In brief, cells were seeded in 96-well plates at a density of 1.0 × 10^5^ cells/well and allowed to attach for 24 h. After incubation with the aforementioned treatments, the culture supernatant was removed and the cells washed with 100 μL/well of 1× assay buffer. The ROS specific stain, DCFH-DA, was added to the cells and allowed to incubate in the dark for 60 min. After the incubation, intracellular ROS level was determined using a fluorescence microplate reader (Ex = 488 nm, Em = 520 nm) (BMG Labtech, Cary, NC, USA).

### 2.11. Statistical Analysis

Data is presented as mean ± S.D. of *n* experiments. Statistical comparisons were performed using t-test or one-way analysis of variance (ANOVA), where appropriate. Differences were considered to be statistically significant at *p* < 0.05. All statistical analysis was performed using GraphPad Prism 5 software (GraphPad Software Inc., San Diego, CA, USA). 

## 3. Results

### 3.1. UPLC-ESI-MS/MS

**Figure 1:**
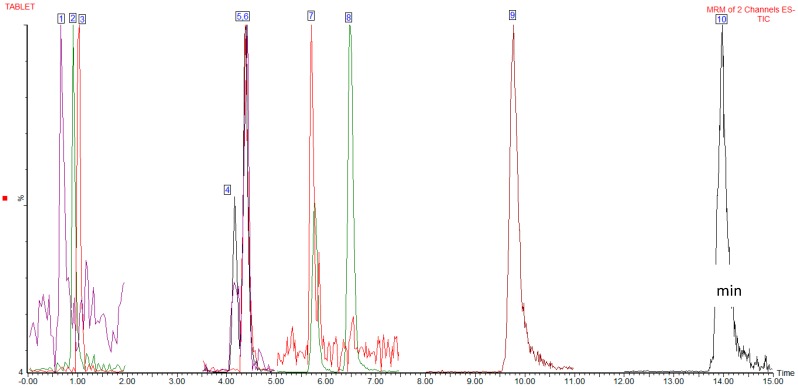
Ultra high-performance liquid chromatography-electrospray ionization-tandem mass spectrometry (UPLC-ESI-MS/MS) chromatogram of NXQ extract.1 = Succinic acid, 2 = 3,4-dihydroxybenzoic acid, 3 = furan-2-carboxylic acid, 4 = quercetin-3-O-galactoside, 5 = quercetin-3-*O*-glucoside, 6 = quercetin-3-*O*-rutinoside, 7 = myricetin, 8 = kaempferol-3-*O*-glucoside, 9 = quercetin, 10 = kaempferol.

The precursor ions for each of the seven analytes and their product ions are shown in Table 2. Comparison of the standard and sample relative ion intensities indicated that all the fragments monitored were within the European Union (EU)-specified tolerances for peak identity confirmation. The ESI voltage is (−) for all the analytes. A representative UPLC-ESI-MS/MS sample chromatogram is shown in Figure 1.

### 3.2. Method Validation Results

The results obtained for the method validation parameters studied are summarised in Table 3. The standard curves indicated good linearity (R^2^ > 0.99) for the analytes. The method had good repeatability. The LOD and LOQ are calculated from 3 times and 10 times the SD of the results for the unspiked analyte sample, respectively. The peak area of some analytes in the sample decreased by >10% at 48 h indicating that the extract should be analysed on the same day it is prepared. Good recoveries and precision of recoveries were obtained for the analytes studied. The recoveries ranged from 84.3–124.3% (mean = 104.2%) and the relative SD (RSD) ranged from 0.5% to 5.3% (mean = 1.8%).

### 3.3. Comparison of Concentrations of the Analytes in Different Batches of NXQ

The concentrations of the analytes tested in 15 batches of NXQ are presented in Table 4. The analytes were present in all the batches tested with the exception of 3,4-dihydroxybenzoic acid where it was not detected in 5 batches (A-5 to 8 and B-1). Myricetin and quercetin-3-*O*-rutinoside were below the LOQ, while succinic acid was below LOD in all batches. There is significant variation in the concentration of the less abundant analytes between the batches with quercetin-3-*O*-galactoside having the greatest variation. The more abundant components showed less variation with quercetin showing the lowest concentration variation.

### 3.4. DPPH and ABTS Scavenging Activity

The readings for DPPH and the ABTS were recorded in triplicate with an average RSD of 3.2 %. The radical scavenging activity of each analyte and sample is reported in Table 5. Kaempferol-3-*O*-glucoside has reported activity in the DPPH assay [27,28,29]. In our assay kaempferol-3-*O*-glucoside had an EC_50_ above 1000 μM, which was beyond the range tested. Yi et al., reported both the activity of quercetin and astragalin, with the quercetin being 18.7 times more active in a cuvette-based DPPH assay [28]. In our assay, this would equate to an approximate EC_50_ for kaempferol-3-*O*-glucoside of 2618 μM which is in agreement with our findings. Park et al., similarly tested kaempferol and kaempferol-3-*O*-glucoside in their DPPH assay, where kaempferol-3-*O*-glucoside was 60 times less active [29]. Astragalin similarly showed some activity in our DPPH assay; however, this did not reach the EC_50_ within the dosage range tested. When the concentration of the analyte is taken into account and their total analyte DPPH activity is taken as a percentage of the NXQ extract, it was found that it only accounts for 20 % of the activity of the whole extract. In terms of corrected DPPH and ABTS activity, the significance of the analytes ranks in the following order: quercetin > kaempferol > quercetin-3-*O*-glucoside > kaempferol-3-*O*-glucoside > quercetin-3-*O*-galactoside > 3,4-dihydroxybenzoic acid > furan-2-carboxylic acid.

### 3.5. Effect of the NXQ Total Extract and the Two Major Components (Quercetin and Kaempferol) of NXQ on the Intracellular ROS Generation in H_2_O_2_-Treated EA.hy926 Cells

**Figure 2:**
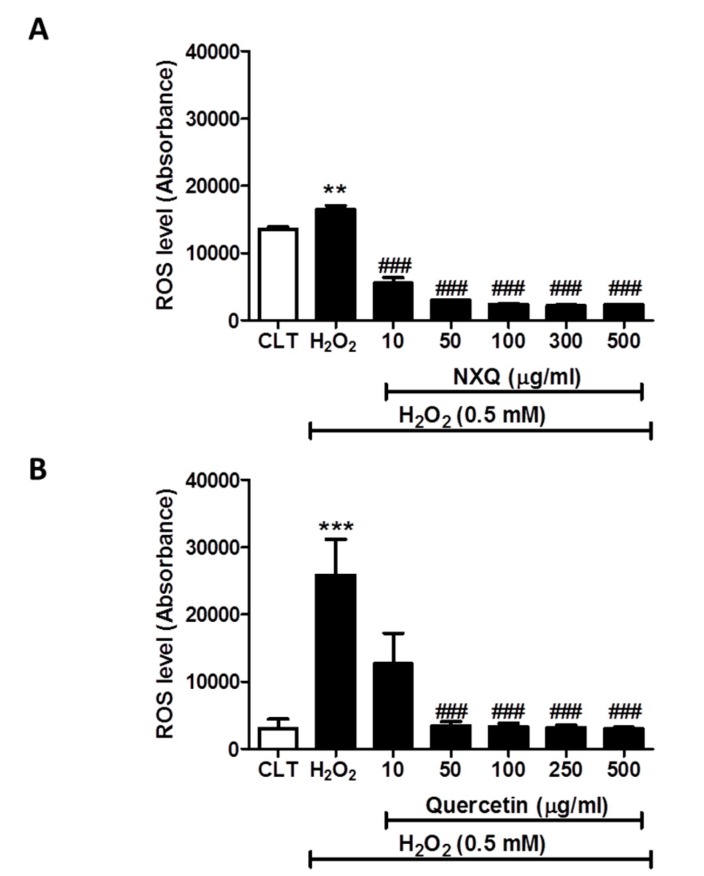

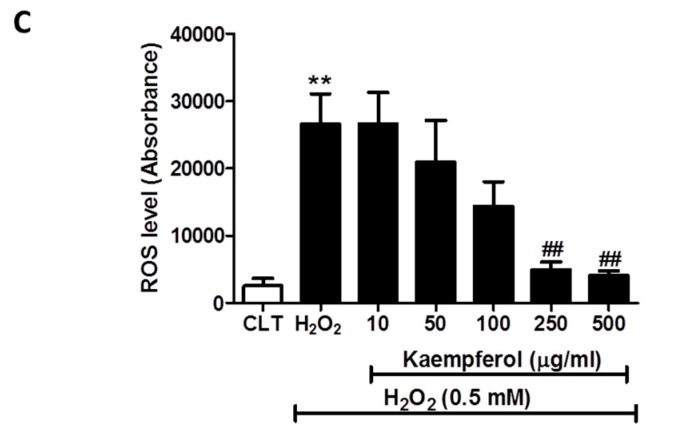
(**A**) Effects of NXQ (10–500 µg mL^−1^) on H_2_O_2_-induced intracellular ROS generation in EA.hy926 cells. (*n* = 3). Data are presented as means ± SD **p <0.001 vs control (CLT) group; ^###^*p* <0.001 vs. H_2_O_2_ group. (**B**) Effects of quercetin (10–500 µg mL^−1^) on H_2_O_2_-induced intracellular ROS generation in EA.hy926 cells. (*n* = 3). Data are presented as means ± S.D. *** *p* <0.0001 vs. control (CLT) group; ^###^*p* <0.001 vs. H_2_O_2_ group. (**C**) Effects of kaempferol (10–500 µg mL^−1^) on H_2_O_2_-induced intracellular ROS generation in EA.hy926 cells. (*n* = 3). Data are presented as means ± SD **p <0.001 vs control (CLT) group; ^##^*p* <0.001 vs. H_2_O_2_ group.

To elucidate the biological activity of the radical scavenging effect of major components in NXQ, the effects on intracellular ROS generation of the total NXQ extract and the top two ranked free radical scavengers in NXQ were evaluated using DCFH-DA (2′,7′-dichlorodihydrofluorescein diacetate), a ROS specific dye. As shown in Figure 2, H_2_O_2_ markedly increased intracellular ROS generation in EA.hy926 cells and NXQ total extract (10–500 µg mL^−1^; *n* = 3) (Figure 2A) and both quercetin (10–500 µg mL^−1^; *n* = 3) (Figure 2B) and kaempferol (10–500 µg mL^−1^; *n* = 4) (Figure 2C) suppressed this H_2_O_2_-induced ROS generation in a concentration-dependent manner. NXQ total extract showed the strongest effect with marked suppression of H_2_O_2_-induced intracellular ROS generation starting at 10 µg mL^−1^. Quercetin significantly inhibited the H_2_O_2_-induced intracellular ROS generation in the range of 50–500 µg mL^−1^, while kaempferol only showed significant inhibition at 250 µg mL^−1^ onward. 

## 4. Discussion

Persimmon tree (*Diospyros kaki*) leaves prepared as a tea has featured in the practice of traditional Chinese medicine for centuries [30]. Their widespread use has resulted in the commercial development of the NXQ tablet containing a standardised extract of this herb. The NXQ extract has been reported to possess various pharmacological properties and has been widely prescribed for the management of a number of cardiovascular diseases such as coronary heart diseases and ischemic stroke. A number of bioactive components, such as flavonoids, biphenyls, polyphenols and triterpenoids, have been identified to be responsible for the therapeutic effects of NXQ [30]. Despite the wide use of NXQ for the management of various cardiovascular diseases, limited work has been done on the chemical analysis of NXQ for quality control purposes. 

To our knowledge, this is the first study to report a validated UPLC-MS/MS method for the analysis of the major bioactive components in NXQ. Previous studies have reported the quantification of five analytes including kaempferol, quercetin, kaempferol-3-*O*-glucoside, furan-2-carboxylic acid and 3,4-dihydroxybenzoic acid in NXQ using separate LC runs [24,31,32,33,34,35]. In this study, we demonstrated the use of a UPLC-MS method for the quantitation of quercetin, kaempferol, kaempferol-3-*O*-glucoside, quercetin-3-*O*-galactoside, quercetin-3-*O*-glucoside, 3,4-dihydroxybenzoic acid, and furan-2-carboxylic acid in NXQ. This is the first publication to report a validated method of analysis for the seven major marker compounds in NXQ in a single run and justify the marker selection. It is important to point out that the traditional high-performance thin layer chromatography profile used for quality assurance is only semi-quantitative and requires considerable operator skill to obtain reproducible results. Utilizing the LC profile as demonstrated in this study provides a more reproducible and accurate quantitation of the target analytes. Furthermore, the use of the MS/MS detector allows for peak identity confirmation by comparing the *m/z* for two product ions (per analyte) and their relative intensities. Given that natural products contain numerous substances/components, peak identity confirmation can greatly reduce the possibility of peak misidentification. 

The relative concentrations of analytes was tested in 15 batches of NXQ. Our result found variation in analyte concentration between the test batches. In instances where dates of manufacture were similar, the difference in analyte concentration between batches may have been due to variation of the chemical composition in the raw material, such as the age of the plant, time of harvest, growth conditions, and post-harvest treatment rather than the manufacturing process. Notwithstanding this, the variation is relatively small for the major active components (e.g., quercetin and kaempferol) when compared to the other minor components. 

Anti-oxidative activity is one of the major pharmacological effects of NXQ for the management of cardio- and cerebrovascular diseases. In this study, we evaluated the anti-oxidant potential of compounds in NXQ to give a measure of their possible contribution to the whole antioxidant capacity. Two radical scavenging assays, DPPH and ABTS assays, were used to assess the abilities of the seven NXQ components on hydrogen atom transfer and electron transfer. These are two possible methods where small molecules can act as an antioxidant [36]. Our results revealed that quercetin and kaempferol ranked as the top two compounds quantified in NXQ in term of abundance and antioxidant activity. In line with that, both quercetin and kaempferol have been reported to be the two main therapeutic constituents in the leaves of *Diospyros kaki L* [15]. Moreover, quercetin is a well-known anti-oxidative compound [37,38,39]. In vitro studies have demonstrated the direct ROS scavenging activity of quercetin [40]. Similarly, the oxidative property of kaempferol has been shown to counteract doxorubicin-induced cardiotoxicity against oxidative stress [41]. Despite significant anti-oxidative activity observed from the compounds quantified in NXQ from this study, when the concentration of the analyte is taken into account and their total analyte DPPH activity is taken as a percentage of the NXQ extract, it is found that it only accounts for 20% of the activity respectively of the whole extract. This finding suggests that the DPPH radical scavenging effect of NXQ is predominately occurring from unidentified compounds or possibly from synergistic interactions. The biological antioxidant effects of NXQ may involve other mechanisms. For example, although the in vitro study has demonstrated quercetin can directly scavenge ROS at the concentration range of 5–50 µM, the concentration of quercetin expected to be found in organs (e.g., heart, pancreas and brain) are likely to be in nanomolar [42] levels, suggesting quercetin can modulate oxidative stress via alternative mechanisms. Indeed, both cell and animal-based studies have demonstrated that quercetin has multiple mechanisms against cellular oxidative stress, such as up regulation of cellular antioxidant enzyme activity and reduction of lipid peroxidation [43,44]. In the current study, we used cultured human vascular endothelial cells, EA.hy926, to evaluate the biological relevance of the top two ranked free radical scavengers qualified in NXQ. Both quercetin and kaempferol significantly reduced the H_2_O_2_-induced ROS generation in EA.hy926 cells in a concentration-dependent manner. Similarly, the NXQ total extract also reduced intracellular ROS generation caused by H_2_O_2_ insult. NXQ total extract showed the strongest anti-oxidative effects followed by quercetin and kaempferol. These results demonstrated that the cellular anti-oxidative activity of NXQ is at least partly mediated by both the quercetin and kaempferol.

In summary, we established a validated UPLC-MS/MS method for the analysis of the major bioactive components in NXQ. This analytical method would enable the medication to be manufactured to a consistent quality in terms of it containing target analytes within a specified concentration range to ensure the medication has the desired physiological effect for the consumer. The analytical method has good precision and accuracy and its performance characteristics are well characterized. Although the detailed underlying mechanism of NXQ in vascular protection remains to be investigated, the pharmacological and biological data obtained from this study clearly demonstrated that the anti-oxidative activity of NXQ and the effects are closely associated with the free radical scavenger activity and inhibition of intracellular ROS generation by quercetin and kaempferol. 

## Figures and Tables

**Table 1:** Set of cone collision energies for each analyte in the electrospray ionization-tandem mass spectrometry (ESI-MS/MS).

Analytes	Energy for the Fragmentation (V)	Retention Time Window (min)
Kaempferol-3-*O*-glucoside	30	5–7.5
Quercetin-3-*O*-galactoside	30	3.5–5
Quercetin-3-*O*-glucoside	35	3.5–5
Kaempferol	40	12–15
3,4-Dihydroxybenzoic acid	15	0–2
Furan-2-carboxylic acid	10	0–2
Quercetin	20	8–11

**Table 2:** The precursor and product ions monitored for each analyte and their relative ion intensities.

Analytes	*m*/*z* Ions		Relative Intensity (%)		
	Precursor [M^−^]	Product	Standard Peak ^1^	Sample Peak ^1^	Relative Difference ^2^
Kaempferol-3-*O*-glucoside	447.75	285.05	100	100	0.00
		255.93	27.3	26.94	1.32
Quercetin-3-*O*-galactoside	463.51	300.95	100	100	0.00
		271.95	4.59	4.39	4.36
Quercetin-3-*O*-glucoside	463.7	301.07	100	100	0.00
		271.88	30.16	30.91	2.49
Kaempferol	285.22	159.04	64.49	80.71	2.85
		116.98	100	100	0.00
3,4-Dihydroxybenzoic acid	153.89	110.03	100	100	0.00
		109.02	24.37	12.37	4.92
Furan-2-carboxylic acid	111.89	67.88	100	100	0.00
		66.81	18.32	17	7.21
Quercetin	301.42	178.82	42.93	39.9	7.06
		150.88	100	100	0.00
Quercetin-3-*O*-rutinoside^3^	609.08	299.86	100	100	0
		271.02	0.91	4.93	45.05
Succinic acid^4^	118	99.83	10.04	7.55	24.80
		73.95	100	100	0
Myricetin^3^	317.66	178.95	74.97	70.22	6.34
		150.94	100	100	0

^1^ Average intensity of the fragments taken from the chromatographic peaks; ^2^ Relative difference = ((standard peak intensity-sample peak intensity)/standard peak intensity) × 100; ^3^ Below limit of quantification (LOQ) in validation sample; ^4^ Below limit of detection (LOD) in validation sample.

**Table 3:** Validation parameters for the seven analytes selected for the analysis of NXQ.

Analytes	R^2 *1*^	Concentration (mg g^−1^) ± SD^2^	LOD/LOQ (mg g^−1^) *^3^*	Recovery (%) (±RSD) *^5^*			
				50 % Spike	100 % Spike	200 % Spike	Average %
Kaempferol-3-*O*-glucoside	0.9998	3.12 ± 0.21	0.6/2.1	92.8 ± 0.8	111.0 ± 1.6	124.3 ± 0.5	109.4 ± 1.0
Quercetin-3-*O*-galactoside	0.9990	1.68 ± 0.14	0.4/1.4	84.3 ± 0.5	118.0 ± 1.6	94.7 ± 1.9	99.0 ± 1.3
Quercetin-3-*O*-glucoside	0.9998	1.65 ± 0.18	0.6/1.8	88.6 ± 1.4	99.9 ± 1.4	117.1 ± 0.7	101.9 ± 1.2
Kaempferol	0.9996	2.73 ± 0.15	0.5/1.5	90.4± 0.8	104.2 ± 2.6	119.7 ± 0.9	104.8 ± 1.4
3,4-Dihydroxybenzoic acid	0.9963	0.52 ± 0.13	0.4/1.3	107.4 ± 1.7	121.8 ± 2.4	95.4 ± 1.1	108.2 ± 1.7
Furan-2-carboxylic acid	0.9987	5.19 ± 0.10	0.3/1.0	101.2 ± 5.3	106.7 ± 3.8	114.9 ± 4.2	107.6 ± 4.4
Quercetin	0.9999	6.43 ± 0.35	1.1/3.5	95.6 ± 1.3	96.5 ± 1.9	104.5 ± 1.2	98.8 ± 1.5

*^1^* R^2^ = linear correlation coefficient *^2^* Seven replicates injected in triplicate on consecutive days *^3^* LOD = limit of detection (determined from 3× SD), LOQ = limit of quantification (determined from 10× SD) *^5^* Calculated from 7 replicates.

**Table 4:** Mean concentration (mg g^−1^) ^1^ of the seven analytes in 15 batches of NXQ pills determined by UPLC-ESI-MS/MS ^1^.

Batch	Kaempferol-3-*O*-glucoside	Quercetin-3-*O*-galactoside	Quercetin-3-*O*-glucoside	Kaempferol	3,4-Dihydroxybenzoic acid	Furan-2-carboxylic acid	Quercetin	DOM ^2^
A-1	3.99	0.83	2.54	2.71	0.63	4.76	3.35	5/1/2011
A-2 ^4^	3.12	1.68	1.65	2.73	0.52	5.19	6.43	8/1/2011
A-3	1.85	3.80	0.50	3.61	0.47	2.78	2.28	11/1/2011
A-4	3.05	0.95	1.79	2.49	1.01	4.80	4.18	7/5/2011
A-5	1.14	0.23	0.48	1.05	<LOD	5.29	4.39	5/6/2011
A-6	1.02	0.10	0.43	1.00	<LOQ	5.57	6.55	24/6/2011
A-7	1.05	0.27	0.34	0.96	<LOQ	1.46	3.54	2/8/2011
A-8	0.82	0.20	0.24	0.89	<LOQ	3.92	4.03	17/8/2011
B-1	1.27	0.39	0.69	1.15	<LOQ	2.44	3.13	11/1/2012
B-2	1.65	0.30	0.87	1.47	<LOQ	3.57	5.28	19/5/2012
B-3	1.86	0.28	0.99	1.54	<LOQ	3.68	5.48	20/5/2012
B-4	1.56	0.31	0.79	1.29	<LOQ	3.64	4.30	21/5/2012
B-5	1.68	0.49	0.92	1.35	<LOQ	3.41	4.51	26/5/2012
B-6	1.47	0.13	0.79	1.32	<LOQ	3.14	5.87	28/5/2012
B-7	1.45	0.45	0.82	1.32	<LOQ	3.53	4.10	29/5/2012
Fold variation ^3^	4.87	36.61	10.58	4.08	33.67 ^5^	3.82	2.87	

*^1^* Mean value calculated from 5 replicates. *^2^* DOM = date of manufacture, organized from oldest to newest. Each batch has an expiry date of 3 years from date of manufacture. *^3^* Fold variation = maximum concentration/minimum concentration. *^4^* Method validation carried out on this batch. ^5^ Zero value omitted for this calculation.

**Table 5:** Radical scavenging activity of each analyte and sample.

Rank	Analyte	DPPH Activity (EC_50_ µM ± SD)	ABTS Activity (EC_50_ µM ± SD)	Average Concentration (mg g^−1^)	Ranking Score ^1^
1	Quercetin	140 ± 2	81 ± 2	4.49	100
2	Kaempferol	119 ± 6	80 ± 3	1.66	37
3	Quercetin-3-*O*-glucoside	148 ± 2	68 ± 3	0.92	21
4	Kaempferol-3-*O*-glucoside	>1000	164 ± 5	1.80	18
5	Quercetin-3-*O*-galactoside	175 ± 5	90 ± 1	0.69	15
6	3,4-Dihydroxybenzoic acid	181 ± 10	350 ± 13	0.20	4
7	Furan-2-carboxylic acid	>1000	>1000	3.81	0

^1^ Ranking score was calculated as the average of DPPH and ABTS activity, multiplied by the concentration, normalized to 100.
